# LncRNA GAS5 contributes to lymphatic metastasis in colorectal cancer

**DOI:** 10.18632/oncotarget.13384

**Published:** 2016-11-16

**Authors:** Yongbin Zheng, Dan Song, Kuang Xiao, Cao Yang, Yu Ding, Wenhong Deng, Shilun Tong

**Affiliations:** ^1^ Department of Gastrointestinal Surgery, Renmin Hospital of Wuhan University, Wuhan, Hubei, PR China

**Keywords:** colorectal cancer, GAS5, metastasis, LncRNA

## Abstract

Colorectal cancer (CRC) ranks the third most common type of cancer worldwide. However, the detailed molecular mechanisms underlying these processes are poorly understood. Recent studies have shown that lncRNAs play important roles in carcinogenesis and progression of CRC. The lncRNA growth arrest special 5 (GAS5), was previously identified to be down-regulated and functions as a tumor suppressor gene in many kinds of cancers. In current two-stage, case-control study, we systematically evaluated the potential role of lncRNA GAS5 and its genetic variation rs145204276 in the development and metastasis process of CRC in a Chinese population. We found the allele del of rs145204276 was significantly associated with 21% decreased risk of CRC (OR=0.79; 95% CI=0.70-0.89; P value = 5.21×10^−5^). Compared with the genotype ins/ins, both the genotype ins/del (OR=0.78; 95% CI=0.68-0.91) and del/del (OR=0.64; 95% CI=0.49-0.84) showed decreased susceptibility. For both in colon and rectum cancers, the associations kept statistically significant (OR=O.78 and 0.80, while P value = 4.56×10^−4^, and 3.80×10^−3^, respectively). The results also showed that the carriers of allele del are less likely to get lymph node metastasis (OR=0.80; 95% CI=0.68-0.95; P value = 0.010). Taken together, our findings provided strong evidence for the hypothesis that GAS5 rs145204276 were significantly associated with the susceptibility and progression of CRC.

## INTRODUCTION

Colorectal cancer (CRC) remains one of the leading cause of cancer-related death and the third most commonly diagnosed cancer in males and the second in females, with an estimated 1.4 million cases and 693,900 deaths occurring in 2012 worldwide [1, 2]. In united States, the estimated new CRC cases and deaths were 134,490 and 49,190 respectively in 2016 [3]. While in China, the estimated new cancer cases and deaths were 376.3 and 191.0 thousands respectively, according to the Chinese National Office for Cancer Prevention and Control [4]. About 11.0% of CRC patients had synchronous lung metastases, which caused that the 3-year relative survival rate was 11.3% [5]. All evidence above suggest that CRC is still a big threat to human health and public health problem. Although some genetic factors, body weight, physically activity, consumption of red and processed meat and alcohol, and smoking has been identified to be associated with the outcomes of CRC patients, relapse and metastasis happened in many CRC patients, however, the detailed molecular mechanisms underlying these processes are poorly understood [6–8].

Increasing evidence shows that long noncoding RNAs (lncRNAs) are involved in all aspects of cellular physiology critical for cancer initiation, progression, and metastasis [9–23]. The lncRNA growth arrest special 5 (GAS5), which was located at 1q25, was identified to be down-regulated and functions as a tumor suppressor gene in many kinds of cancers, including breast cancer, prostate cancer, pancreatic cancer, bladder cancer, lung cancer, gastric cancer, and so on [24–31]. Tao et al [32] also identified that the deletion allele of a 5-base pair indel polymorphism (rs145204276) in the promoter region of GAS5 significantly increased the risk of hepatocellular carcinoma (HCC) and increased the expression of GAS5 in hepatocellular cell lines, which indicated the differential roles of GAS5 in carcinogenesis of different cancer types. In current study, we aim to systematically evaluate the potential role of lncRNA GAS5 and its genetic variation rs145204276 in the development and metastasis process of CRC in a Chinese population.

## RESULTS

### Population characteristics

As shown in Table 1, characteristics of the subjects included in the two stage of current study were generally comparable. No significant difference were detected for age group, gender, alcohol status and smoking status between CRC cases and healthy controls (all the P value > 0.05). Table 1 also present the percentages of different tumor sites (Colon and Rectum), lymph node metastasis and distant metastasis, which are similar between the two stages in current study. The distribution of genotypes of rs145204276 in healthy controls in the two stage was in accordance with Hardy-Weinberg equilibrium (HWE, P > 0.05).

**Table 1:** The characteristics of the study population

Variables	Stage I			Stage II		
	Cases (n=600)	Controls (n=600)	P value	Cases (n=800)	Controls (n=800)	P value
Age group						
≥60	255 (42.5%)	264 (44.0%)	0.600	365 (45.6%)	362 (45.2%)	0.880
<60	345 (57.5%)	336 (56.0%)		435 (54.4%)	438 (54.8%)	
Gender						
Male	369 (61.5%)	372 (62.0%)	0.859	480 (60.0%)	468 (58.5%)	0.542
female	231 (38.5%)	228 (38.0%)		320 (40.0%)	332 (41.5%)	
Smoking status						
Smokers	186 (31.0%)	171 (28.5%)	0.344	232 (29.0%)	212 (26.5%)	0.264
Non-Smokers	414 (69.0%)	429 (71.5%)		568 (71.0%)	588 (73.5%)	
Alcohol status						
drinkers	201 (33.5%)	180 (30.0%)	0.193	280 (35.0%)	256 (32.0%)	0.204
Non-drinkers	399 (66.5%)	420 (70.0%)		520 (65.0%)	544 (68.0%)	
Tumor site						
Colon	340 (56.7%)			466 (58.2%)		
Rectum	260 (43.3%)			334 (41.8%)		
Lymph node metastasis						
No	390 (65.0%)			500 (62.5%)		
Yes	210 (35.0%)			300 (37.5%)		
Distant metastasis						
No	507 (84.5%)			688 (86.0%)		
Yes	93 (15.5%)			112 (14.0%)		

### Associations between GAS5 rs145204276 and CRC susceptibility

Table 2 presents the association between GAS5 rs145204276 and CRC susceptibility in two independent stages. In stage I, the allele del was significantly associated with decreased risk of CRC (OR=0.81; 95% CI=0.68-0.96; P value = 0.016). Thus, we replicated the association in an independent stage (stage II), which also presented a significant association (OR=0.78; 95% CI=0.67-0.91; P value = 1.24×10^−3^). When pooled together, the allele del was significantly associated with 21% decreased risk of CRC (OR=0.79; 95% CI=0.70-0.89; P value = 5.21×10^−5^). Compared with the genotype ins/ins, both the genotype ins/del (OR=0.78; 95% CI=0.68-0.91) and del/del (OR=0.64; 95% CI=0.49-0.84) showed decreased susceptibility.

**Table 2:** Associations between GAS5 rs145204276 and CRC susceptibility among Chinese population

Genotypes	Cases (n, %)	Controls (n, %)	OR (95% CI)^a^	P_trend_
**Stage I**				
ins/ins	320 (53.3%)	279 (46.5%)	1.00 (Reference)	
ins/del	230 (38.3%)	258 (43.0%)	0.78 (0.61-0.99)	
del/del	50 (8.4%)	63 (10.5%)	0.69 (0.46-1.03)	
del vs ins			0.81 (0.68-0.96)	**0.016**
**Stage II**				
ins/ins	418 (52.3%)	360 (45.0%)	1.00 (Reference)	
ins/del	320 (40.0%)	352 (44.0%)	0.78 (0.64-0.96)	
del/del	62 (7.7%)	88 (11.0%)	0.61 (0.43-0.86)	
del vs ins			0.78 (0.67-0.91)	**1.24×10^−3^**
**Total effect**				
ins/ins	738 (52.7%)	639 (45.6%)	1.00 (Reference)	
ins/del	550 (39.3%)	610 (43.6%)	0.78 (0.68-0.91)	
del/del	112 (8.0%)	151 (10.8%)	0.64 (0.49-0.84)	
del vs ins			0.79 (0.70-0.89)	**5.21×10^−5^**

a adjusted by age, gender, alcohol and smoking status

### Associations between GAS5 rs145204276 and CRC susceptibility stratified by Tumor site

Then the associations between GAS5 rs145204276 and CRC susceptibility were analyzed stratified by Tumor site (Table 3). For both in colon and rectum cancers, the associations kept statistically significant (OR=O.78 and 0.80, while P value = 4.56×10^−4^, and 3.80×10^−3^, respectively).

**Table 3:** Associations between GAS5 rs145204276 and CRC susceptibility stratified by Tumor site

Genotypes	Cases (n, %)	Controls (n, %)	OR (95% CI)^a^	P_trend_
**Colon**				
ins/ins	428 (53.1%)	639 (45.6%)	1.00 (Reference)	
ins/del	313 (38.8%)	610 (43.6%)	0.77 (0.64-0.92)	
del/del	65 (8.1%)	151 (10.8%)	0.64 (0.47-0.88)	
del vs ins			0.78 (0.68-0.90)	**4.56×10^−4^**
**Rectum**				
ins/ins	310 (52.2%)	639 (45.6%)	1.00 (Reference)	
ins/del	237 (39.9%)	610 (43.6%)	0.80 (0.65-0.98)	
del/del	47 (7.9%)	151 (10.8%)	0.64 (0.45-0.91)	
del vs ins			0.80 (0.69-0.93)	**3.80×10^−3^**

a adjusted by age, gender, alcohol and smoking status

### Associations between GAS5 rs145204276 and Lymph node metastasis and Distant metastasis of CRC

To determine whether GAS5 rs145204276 can affect the progression of CRC, we also explored the associations between GAS5 rs145204276 and Lymph node metastasis and Distant metastasis of CRC. As shown in Table 4, the carriers of allele del are less likely to get lymph node metastasis (OR=0.80; 95% CI=0.68-0.95; P value = 0.010). Compared with those owing the genotype ins/ins, both subjects with the genotype ins/del (OR=0.86; 95% CI=0.68-1.08) and genotype del/del (OR=0.58; 95% CI=0.37-0.90) showed decreased possibility of lymph node metastasis. Due to limited the sample size and statistical power, the association between GAS5 rs145204276 and distant metastasis of CRC was marginal significant (OR=0.80; 95% CI=0.63-1.02; P value = 0.07).

**Table 4:** Associations between GAS5 rs145204276 and Lymph node metastasis and Distant metastasis of CRC

Genotypes	Event (n, %)	No event (n, %)	OR (95% CI)^a^	P_trend_
**Lymph node metastasis**				
ins/ins	286 (56.1%)	452 (50.8%)	1.00 (Reference)	
ins/del	194 (38.0%)	356 (40.0%)	0.86 (0.68-1.08)	
del/del	30 (5.9%)	82 (9.2%)	0.58 (0.37-0.90)	
del vs ins			0.80 (0.68-0.95)	**0.010**
**Distant metastasis**				
ins/ins	117 (57.1%)	621 (52.0%)	1.00 (Reference)	
ins/del	78 (38.0%)	472 (39.5%)	0.88 (0.64-1.20)	
del/del	10 (4.9%)	102 (8.5%)	0.52 (0.27-1.02)	
del vs ins			0.80 (0.63-1.02)	0.070

a adjusted by age, gender, alcohol and smoking status

### Relative expression of GAS5

**Figure 1:**
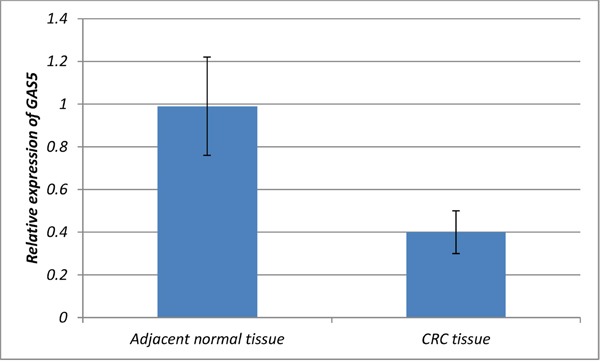
Relative expression of GAS5

we also examined expression level of GAS5 in CRC tumor tissues and adjacent normal tissues of 50 CRC cases (Figure 1). Among all the pairs of CRC patients, the expression levels of lncRNA GAS5 in CRC tissues were significantly lower than those in the corresponding normal tissues (P<0.001).

## DISCUSSION

In this two-stage, case-control study with large sample size, we systematically evaluated the association of GAS5 rs145204276 with the susceptibility and progression of CRC among Chinese population. We found the allele del of rs145204276 was significantly associated with 21% decreased risk of CRC (OR=0.79; 95% CI=0.70-0.89; P value = 5.21×10^−5^). The associations kept statistically significant in colon and rectum cancers, and the results also showed that the carriers of allele del are less likely to get lymph node metastasis. To the best of our knowledge, this should be first study which systematically evaluated the genetic association of GAS5 with the susceptibility and progression of CRC.

LncRNAs have been identified to be involved in multiple biological functions, and also plays a vital role in CRC carcinogenesis [15, 33–48]. Some lncRNAs are related to the poor prognosis of CRC [39, 42, 44, 45], while some others are associated with occurrence of CRC [33, 37, 43, 47]. Han et al [48] also found that 14 lncRNAs were specifically up-regulated and 5 specifically down-regulated in metastatic lymph node of CRC patients, compared with those of normal lymph node. Recently, Chen et al [34] identified that 2636 lncRNAs, including 1600 up-regulated and 1036 down-regulated over two-fold compared with the CRC tissues without metastasis, were associated with liver metastasis of CRC through a genome-wide analysis of lncRNA expression. All studies above provide solid evidence that lncRNAs paly essential role in the development and metastasis process of CRC.

The GAS5 gene, which was first reported by Coccia et al in 1992, was isolated from mouse genomic DNA and structurally characterized [49]. The transcriptional unit is divided into 12 exons that span around 7 kb [49]. Then Nakamura et al [50] found that The GAS5 gene fuses to BCL6 as a result of t(1;3)(q25;q27) in a patient with B-cell lymphoma. Also GAS5, whose transcript levels were significantly reduced in breast cancer samples relative to adjacent unaffected normal breast epithelial tissues, could controls apoptosis and down-regulated in breast cancer [31]. Further literatures reported the potential role of GAS5 in the human T-lymphocytes, renal cell carcinoma, prostate cancer, pancreatic cancer, bladder cancer, non-small-cell lung cancer, gastric cancer, hepatocellular carcinoma, cervical cancer, and so on [27–30, 51–55]. Yin et al [56] found that GAS5 could also affects cell proliferation and predicts a poor prognosis in patients with CRC, although the sample size of recruited patients were limited (only 66 CRC patients).

In current study, we found GAS5 rs145204276 was associated with not only the susceptibility of CRC, but also the lymph node metastasis of CRC. SNP rs145204276 was located in the promoter region of GAS5, and luciferase activity analysis suggested that the deletion allele improved an increased expression of GAS5 [32]. In current study, the statistical power for such an association was 98.1%. Using the online database RegulomeDB (http://regulomedb.org), we found that rs145204276 could bind protein POLR2A, MAX, GATA1, BHLHE40, FOXP2, ATF3, USF2, and so on. While HaploReg v4.1 (http://www.broadinstitute.org/mammals/haploreg/haploreg.php) found that rs145204276 could alter the 9 regulatory motifs, including E2F_disc3, EWSR1-FLI1, MZF1::1-4_3, PTF1-beta, Pbx3_disc3, SP1, STAT, UF1H3BETA, and Znf143.

Conclusively, in this two-stage, case-control study integrating bioinformatics analysis and large-sample-size, we highlighted a potential functional locus, GAS5 rs145204276, for susceptibility and progression of CRC. Systematic researches on different population and more susceptibility loci are warranted to identify causal variants and elaborate the genetic etiology for susceptibility and progression of CRC.

## MATERIALS AND METHODS

### Subjects

This study was a two-stage, case–control sets including 600 newly diagnosed incident CRC cases and 600 healthy controls in stage I, and 800 newly diagnosed incident CRC cases and 800 healthy controls in stage II, which were recruited between 2010 and 2015. All patients had never received any medical treatments, and the diagnosis of patients was validated through pathologic examination by two different senior pathologists. Healthy controls free of any type cancers were selected from health check-up programs at the same hospital during the same period. Then they were matched to the CRC cases by gender and age group, gender, alcohol and smoking status. Then, 5 ml peripheral blood was collected from each subject, and demographic and pathological information were face to face collected by interviewers. The study was approved by appropriate Research Ethics Committee (REC), and written informed consent was obtained from all patients.

### Genotyping

Extraction of the genomic DNA from blood samples and HCC tumor tissues was conducted using Qiagen genomic DNA purification kit. DNA fragments containing the indel polymorphism were amplified using the following genotyping primers: F-TCCCGACTGAGGAGGAAGAGCA; R-AACACC GTCCCGGAAGTGAAA. The PCR products were analyzed by 7% non-denaturing polyacrylamide gel electrophoresis and visualized by silver staining. Quality control was performed by direct sequencing 5% duplicate samples in blind, with a concordance rate of 100%. Furthermore, a 5% random selected sample was tested in duplicate by different persons, and the concordance rate was 100%.

### Statistical analyses

Two-sided Student's t-test was selected to compare the differences in the quantitative data, while χ2-test was used to analyze the differences of categorical data between the two groups. Odds ratios (ORs) and 95% confidence intervals (95% CIs) were selected to estimate the strength of association between rs145204276 and risk of CRC and its Lymph node metastasis and Distant metastasis by unconditional multivariable logistic regression, adjusted by age group, gender, alcohol and smoking status. HWE of genotypes was evaluated in controls by a goodness-of-fit χ^2^ test. All statistics were performed using SPSS software 19.0 (SPSS Inc., Chicago, IL, USA), and P values were two sided with the statistical significance criteria of P < 0.05 all through the study.
